# Expression of human *Piwi-like* genes is associated with prognosis for soft tissue sarcoma patients

**DOI:** 10.1186/1471-2407-12-272

**Published:** 2012-06-29

**Authors:** Thomas Greither, Franziska Koser, Matthias Kappler, Matthias Bache, Christine Lautenschläger, Steffen Göbel, Hans-Jürgen Holzhausen, Sven Wach, Peter Würl, Helge Taubert

**Affiliations:** 1 Center for Reproductive Medicine and Andrology, Martin-Luther-University Halle-Wittenberg, Halle, Germany; 2 Department of Oral and Maxillofacial Plastic Surgery, Martin-Luther-University Halle-Wittenberg, Halle, Germany; 3 Department of Radiotherapy, Martin-Luther-University Halle-Wittenberg, Halle, Germany; 4 Institute of Medical Biometry and Informatics, Martin-Luther-University Halle-Wittenberg, Halle, Germany; 5 Institute of Transfusion Medicine, Martin-Luther-University Halle-Wittenberg, Halle, Germany; 6 Institute of Pathology, Martin-Luther-University Halle-Wittenberg, Halle, Germany; 7 Div. Molecular Urology, FAU Erlangen-Nürnberg, University Clinic of Urology, Erlangen, Germany; 8 Nikolaus-Fiebiger-Center for Molecular Medicine, FAU Erlangen-Nürnberg, Erlangen, Germany; 9 Department of General and Visceral Surgery, Diakoniekrankenhaus Halle, Halle, Germany; 10 Klinik für Urologie und NFZ, FAU Erlangen, Glückstr. 6, D-91054, Erlangen, Germany

## Abstract

**Background:**

Argonaute genes are essential for RNA interference, stem cell maintenance and differentiation. The *Piwi-like* genes, a subclass of the Argonaute genes, are expressed mainly in the germline. These genes may be re-expressed in tumors, and expression of the *Piwi-like* genes is associated with prognosis in several types of tumors.

**Methods:**

We measured the expression of *Piwi-like* mRNAs (*Piwi-like 2*–*4*) in 125 soft tissue sarcoma (STS) samples by qPCRs. Statistical tests were applied to study the correlation of expression levels with tumor-specific survival for STS patients.

**Results:**

In multivariate Cox’s regression analyses, we showed that low *Piwi-like 2* and *Piwi-like 4* mRNA expression were significantly associated with a worse prognosis (RR = 1.87; p = 0.032 and RR = 1.82; p = 0.039). Low expression of both genes was associated with a 2.58-fold increased risk of tumor-related death (p = 0.01). *Piwi-like 4* and combined *Piwi-like 2* and *4* mRNA levels correlated significantly with prognosis (RR = 3.53; p = 0.002 and RR = 5.23; p = 0.004) only for female but not for male patients. However, combined low *Piwi-like 2* and *3* transcript levels were associated with worse survival (RR = 5.90; p = 0.02) for male patients.

**Conclusions:**

In this study, we identified a significant association between the expression of *Piwi-like 2* and *4* mRNAs and the tumor-specific survival of soft tissue sarcoma patients. Furthermore, a connection between sex and the impact of *Piwi-like* mRNA expressions on STS patients’ prognosis was shown for the first time.

## Background

The Piwi (P-element-induced wimpy testis) family is a subclass of the Argonaute gene/protein family characterized by their homology and the occurrence of PAZ and Piwi domains [1,2]. PIWI proteins play important roles in stem cell self-renewal, spermatogenesis, transposon and RNA silencing, translational regulation, and chromatin remodeling in various organisms [3]. Cox and coworkers identified the first Piwi gene in Drosophila [4]. Piwi is required for the asymmetric division of germ-line stem cells (GSCs) to produce and to maintain a daughter GSC (stem cell self-renewal). In addition, homologues of the *Piwi* gene have been identified in *Caenorhabditis elegans, Arabidopsis* and humans, but not in bacteria or yeast, suggesting that Piwi has a stem cell-related function existing only in multicellular organisms. *Piwi-like* genes also play a role in adult somatic stem cells, such as human hematopoietic stem cells and mouse mesenchymal stem cells [5,6]. Four members of the human *Piwi *gene family, *Piwi-like 1* (*Hiwi, Piwil1*), *Piwi-like 2* (*Hili, Piwil2*), *Piwi-like 3* (*Piwil3*) and *Piwi-like 4* (*Hiwi2, Piwil4*), have been described so far [7]. The genes have been mapped to different chromosomal regions. *Piwi-like 1* and *Piwi-like 2* are located on chromosome 12 (12q23-12q24.33) and chromosome 8 (8p21). *Piwi-like 3* and *Piwi-like 4* have been mapped to chromosome 22 (22q11.2) and chromosome 11 (11q12).

An increasing number of publications have demonstrated that members of the Piwi family may be re-expressed in malignant tumors. Expression of these genes may correlate with tumor behavior and patient prognosis. Elevated protein expression of PIWIL1 is associated with a poor prognosis in seminoma, gastric cancer, esophageal cancer and glioma [8-11]. PIWIL2 protein may be detected in various stages of cervical squamous cell carcinoma and breast adenocarcinoma, as well as in metaplastic epithelial cells and histologically normal-appearing tissues adjacent to breast and cervical tumors [12-14]. Additionally, high levels of PIWIL2, PIWIL3 and PIWIL4 proteins are correlated with an elevated risk of colon cancer [15]. Li and coworkers also showed that increased PIWIL4 protein levels are significantly associated with the risk of metastasis and prognosis in colon cancer patients [15].

Protein levels and mRNA levels are not necessarily correlated [16]. Reasons for this could be different mRNA stability, mRNA degradation, and posttranscriptional regulation mechanisms. There are regulatory layers of the transcriptome in which RNA-binding proteins (RBPs), noncoding regulatory RNAs (ncRNAs) and messenger RNAs (mRNAs) can interact [17].

However, there have been comparatively few studies evaluating the impact of *Piwi-like 1–4* mRNA expression in human cancers. In patients with gastric cancer and soft tissue sarcoma (STS) and in male pancreatic carcinoma patients, the levels of mRNA expression of *Piwi-like 1* are correlated with prognosis [9,18,19]. Expression of the *Piwi-like* 2 gene has been detected in many different human tumors, including prostate, breast, gastrointestinal, ovarian and endometrial cancers [20]. However, no data have been published about *Piwi-like 3* and *Piwi-like 4* mRNA expression levels in human tumors. A search in the Oncomine database revealed an expression of *Piwi-like 3* in carcinomas of the breast, colon, ovary and brain and for *Piwi-like 4* in carcinomas of the breast, liver and brain. In this study, we investigated *Piwi-like* mRNA expression in patients with STS. We attempted to correlate *Piwi-like* mRNA levels with clinical factors such as tumor size and with tumor-specific survival. We also performed a sex-specific analysis of the prognostic impact of *Piwi-like* mRNA expression in patients with STS.

## Methods

### Patients

In this study, tumor tissue samples of 125 patients with STS were analyzed. All patients underwent surgical resection in the Department of Surgery, Martin-Luther-University Halle-Wittenberg and the Department of Surgery 1, University of Leipzig, Germany. The cohort has been described in previous studies [21]. All diagnoses were verified by an experienced pathologist (HJH) according to the UICC system. All patients gave written informed consent. The study was approved by the Ethic Committee of the Medical Faculty of the Martin-Luther-University Halle-Wittenberg and it was performed in compliance with the Helsinki Declaration. Median patient age at the time of surgery was 58 years (range 14–87 years). Median follow-up time was 32 months (range 2–201 months after primary tumor resection). Forty-nine patients experienced locoregional recurrence, 10 patients developed distant metastases and 61 patients died during the observation time. All tumor samples were collected before radio- or chemotherapy. For clinical and histopathologic parameters, please refer to Table 1.

**Table 1:** **The histopathologic, clinical and *Piwi-like *mRNA expression data of STS patients**

**Patients’ characteristics**	**No. of cases**	***Piwi-like 2***		***Piwi-like 3***		***Piwi-like 4***	
		**low**	**high**	**low**	**high**	**low**	**high**
Total	125	62	63	62	63	62	63
Sex							
Male	57	29	28	26	31	27	30
Female	68	33	35	36	32	35	33
Histological subtype							
LS	28	10	18	14	14	11	17
MFH	31	12	19	17	14	19	12
FS	7	4	3	5	2	3	4
RMS	8	5	3	2	6	5	3
LMS	21	14	7	9	12	9	12
NS	12	6	6	4	8	6	6
Syn	10	5	5	7	3	5	5
Other	8	6	2	4	4	4	4
Tumor size^1^							
T1	29	17	12	10	19	18	11
T2	96	45	51	29	67	44	52
Tumor grade							
I	19	7	12	7	12	5	14
II	58	30	28	29	29	31	27
III	48	25	23	26	22	26	22
Tumor stage							
I	16	6	10	7	9	5	11
II	57	28	29	29	28	29	28
III	40	21	19	21	19	22	18
IV	12	7	5	5	7	6	6
Complete resection							
Radical (R0)	87	44	43	44	43	42	45
Not radical (R1)	38	18	20	18	20	20	18
Location							
Extremities	80	36	44	41	39	41	39
Trunk wall	12	9	3	8	4	6	6
Head/neck	4	2	2	1	3	4	0
Abdomen/retroperitoneum	27	14	13	12	15	11	16
Multiple locations	2	1	1	0	2	0	2
Patient status							
Alive	64	26	38	31	33	30	34
Dead	61	36	25	31	30	32	29

Abbreviations: *No*-number of cases, *LS*-liposarcoma, *MFH*-malignant fibrous histiocytoma, *FS*-fibrosarcoma, *RMS*-rhabdomyosarcoma, *LMS*-leiomyosarcoma, *NS*-neuronal sarcoma, *Syn*-synovial sarcoma.

^1^T1 ≤ 5 cm, T2 > 5 cm.

### RNA isolation

Ten to twenty tumor tissue slices (30 μm) were used for RNA isolation with Trizol reagent according to the manufacturer’s protocol (Invitrogen, Karlsruhe, Germany). Briefly, RNA was extracted with phenol/chloroform and precipitated by isopropanol. Any remaining DNA traces were removed by DNAse I digestion (Qiagen, Hilden, Germany). The remaining pellet was washed twice in ice-cold ethanol and resuspended in 30 μl of RNAse-free water. RNA concentration was assessed with a Nanodrop ND-1000 Spectrophotometer (Thermo Scientific, Karlsruhe, Germany). RNA was stored at −80 °C.

### cDNA synthesis

Two micrograms of RNA were used for cDNA synthesis. cDNA was synthesized with the RevertAid H Minus First strand cDNA Synthesis Kit according to the manufacturer’s protocol (Fermentas, St. Leon-Rot, Germany).

### qPCR

*Piwi-like* gene expression was measured using TaqMan Gene expression assays (*Piwi-like 2*: Hs01032719; *Piwi-like 3*: Hs00908837; *Piwi-like 4*: Hs00895218) according to the manufacturer’s protocol (Applied Biosystems, Darmstadt, Germany). The recognition sites of primers within the *Piwi-like* mRNAs are given in Additional file 1: Figure S1. Quantitative RT-PCR reactions were performed with the HotStart-Taq Polymerase Kit (Qiagen, Hilden, Germany) in a real-time cycler (LTF, Wasserburg; Germany). *HPRT* (hypoxanthine phosphoribosyl transferase) expression was measured with the following primers (*HPRT* fw: 5′-TTGCTGACCTGCTGGATTAC-3′; rw: 5′-CTTGCG ACCTTGACCATCTT-3′) using Maxima SYBR-Green qPCR Master Mix (Fermentas, St. Leon-Rot, Germany) and served as reference gene. All measurements were carried out from the same cDNA aliquot within a short time period to ensure comparable conditions. Gene expression was normalized to *HPRT* mRNA expression. Quantification was performed using self-established plasmid standards for *Piwi-like 2, 3* and *4* and *HPRT* in the range of 10^4^–10^8^ copies/μl. Relative mRNA expression ratios (copies of transcript per fg *HPRT*) were used for subsequent statistical analyses.

### Statistical analyses

Bivariate correlation analyses were performed by Spearman rank correlations (r_s_). Kaplan-Meier and multivariate Cox’s regression analyses were performed to analyze the correlation of *Piwi-like 2–4* mRNA expression levels with tumor-specific survival. All statistical analyses were conducted with PASW 18 software (IBM SPSS, Chicago, IL, USA). P-values < 0.05 were considered significant.

## Results

### Expression of *Piwi-like 2–4* mRNAs and their association with clinical, histopathologic and molecular parameters

*Piwi-like 2*, *Piwi-like 3* and *Piwi-like 4* mRNA expression levels were measured in tissue samples from 125 STS patients. Cut-off values were set according to the median value for each gene (*Piwi-like* 2: 7.583 copies/fg *HPRT* (range: 0.073–532.45), *Piwi-like 3*: 0.05 copies/fg *HPRT* (range: 0–52.82) and *Piwi-like 4*: 1.754 copies/fg *HPRT* (range: 0–22.89)).

In addition we studied the *Piwi-like 2*, *Piwi-like 3* and *Piwi-like 4* mRNA expression levels in normal tissue samples adjacent to tumor tissues from 22 out of the 125 STS patients. We detected median mRNA expression levels for *Piwi-like 2*: 0.5 copies/fg *HPRT* (range 0.04–24.84), for *Piwi-like 3*: 0.0 copies/fg *HPRT* (range 0–5), and for *Piwi-like 4*: 0.28 copies/fg *HPRT* (range 0–39.5). The *Piwi-like 2*, *Piwi-like 3* and *Piwi-like 4* mRNA expression levels are higher in the tumor tissues compared to those in the normal tumor adjacent tissues. But they are not correlated with each other (Spearman-Rho test) and the median values of their differences are unequal (p < 0.05; Wilcoxon test).

We performed a bivariate linear correlation analysis (Spearman-Rho-test) to examine whether the mRNA expression levels of individual *Piwi-like* genes were correlated with the mRNA expression levels of the other *Piwi-like* genes in the tumor tissues. The expression levels of mRNA for *Piwi-like 2* and *Piwi-like 4* were highly significantly correlated (r_s_ = 0.36; p = 0.00003), whereas no significant correlation was detected for either mRNA with *Piwi-like 3* in the tumor tissues.

Bivariate linear correlation analysis was also performed to analyze the relationship of *Piwi-like* mRNA expression with clinical factors. The mRNA levels of all *Piwi-like* genes studied were positively correlated to tumor size (*Piwi-like 2*: r_s_ = 0.21; p = 0.018; *Piwi-like 3*: r_s_ = 0.20; p = 0.024; and *Piwi-like 4*: r_s_ = 0.18; p = 0.041), Next, we investigated the correlation of *Piwi-like 2–4* mRNAs with other stem cell-associated genes (*Nanog, Oct3/4* and *survivin*). *Piwi-like 2* mRNA expression was inversely correlated with transcript levels of Oct3/4 and Nanog in our STS cohort (r_s_ = −0.31; p = 0.002 and r_s_ = −0.23; p = 0.029, respectively) but was positively correlated with the splice variant 2B of the* survivin* gene (r_s_ = 0.20; p = 0.048). *Piwi-like 3* mRNA expression was also negatively correlated with *Nanog* transcript levels (r_s_ = −0.24; p = 0.02). *Piwi-like 4* mRNA levels were negatively associated with the expression of the splice variant Delta 3 of the *survivin* gene (r_s_ = −0.18; p = 0.047; Additional file 2: Table S1).

### Association of the *Piwi-like 2–4* mRNA expression level with tumor-specific survival for all STS patients

Multivariate Cox’s regression analyses (adjusted for clinical parameters including resection type, tumor stage, tumor location and tumor type) were performed to study the effect of the mRNA expression level of the three *Piwi-like* genes on tumor-specific survival. For *Piwi-like 2*, low mRNA expression was significantly associated with a worse prognosis (RR = 1.87, 95% CI: 1.06–3.32; p = 0.032). Low *Piwi-like 4* mRNA expression also correlated significantly with decreased tumor-specific survival (RR = 1.82, 95% CI: 1.03–3.21; p = 0.039). *Piwi-like 3* expression was not significantly associated with the prognosis of STS patients (RR = 1.33, 95% CI: 0.75–2.3; p = 0.329).

**Table 2:** **Multivariate Cox’s regression analyses on the impact of *Piwi-like 2, -3 *and − *4 *mRNA expression on STS patients’ survival**

	***Piwi-like 2***	***Piwi-like 3***	***Piwi-like 4***
Relative risk (RR)	**1.87**	1.33	**1.82**
95% CI	1.06–3.32	0.75–2.3	1.03–3.21
p-value	**0.032**	0.329	**0.039**
n low	62	62	62
n high	63	63	63
	Combination *Piwi-like 2* and −*4*	Combination *Piwi-like 2*, -*3 and −4*	
Relative risk (RR)	**2.58**	**4.01**	
95% CI	1.26–5.29	1.61–10.01	
p-value	**0.01**	**0.003**	
n all low	39	21	
n one gene elevated	46	43	
n two genes elevated	–	37	
n all genes elevated	40	24	

Significant p-values and relative risks are marked in bold face (p-values <0.05 were considered significant).

**Figure 1:**
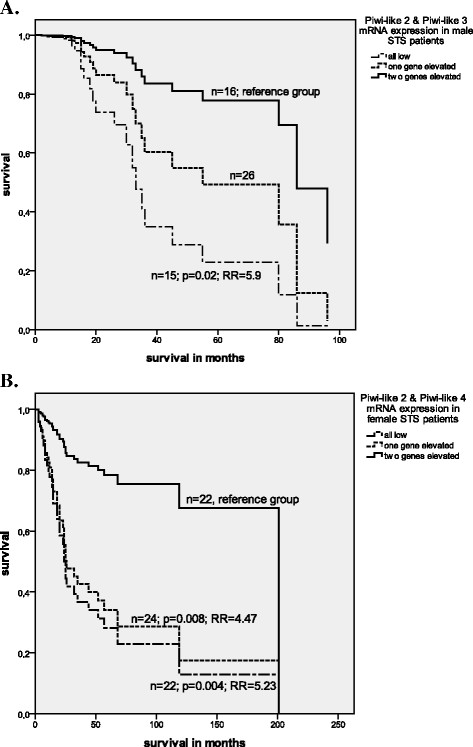
**Multivariate Cox’s regression analyses**: **A**. Combined expression of *Piwi-like 2 and 3* in male STS patients and **B**. combined *Piwi-like 2* and −*4* expression in female STS patients and their correlation with tumor-specific survival.

Next, we studied the combined expression of the *Piwi-like 2 – 4* and their correlation with tumor-specific survival. Patients expressing low levels of either *Piwi-like 2/Piwi-like 3* or of *Piwi-like 2/Piwi-like 4* had a significantly increased risk of tumor-related death compared with patients with a high expression level of the *Piwi-like* mRNAs (RR = 2.10, 95% CI: 1.01–4.40; p = 0.048 and RR = 2.58, 95% CI: 1.26–5.29; p = 0.01, respectively; Table 2 ).

### Association of the *Piwi-like 2–4* mRNA expression level with tumor-specific survival in a sex- specific manner for STS patients

We performed separate analyses of the association of *Piwi-like 2–4* expression in male and female patients (Additional file 3: Figure S2). Female patients with a low level of *Piwi-like 4* mRNA expression in their tumors had a 3.53-fold significantly increased risk of tumor-related death (95% CI: 1.56–8.0; p = 0.002). Furthermore, in females, a trend for increased risk of tumor-related death was seen in patients with low levels of *Piwi-like 2* expression (RR = 1.88, 95% CI: 0.87–4.07). However, this result was not statistically significant (p = 0.106). Male patients with low levels of *Piwi-like 2* or *Piwi-like 3* had a 2.76-fold and a 2.59-fold increased risk of tumor-related death. However, this result was also not statistically significant (Table 3).

**Table 3:** **Sex-specific association between *Piwi-like 2–4 *mRNA expression and STS patient survival**

	**Female patients**				**Male patients**			
	***Piwi-like 2***	***Piwi-like 3***	***Piwi-like 4***		***Piwi-like 2***	***Piwi-like 3***	***Piwi-like 4***	
Relative risk (RR)	1.88	1.13	**3.53**		2.76	2.59	1.10	
95% CI	0.87–4.07	0.51–2.52	1.56–8.00		0.95–8.04	0.81–8.33	0.36–3.31	
p-value	0.106	0.757	**0.002**		0.062	0.11	0.87	
n low	34	34	34		28	28	28	
n high	34	34	34		29	29	29	
	**Combination *Piwi-like 2 *and − *4***	**Relative risk (RR)**	**95**% **CI**	**p-value**	**Combination *Piwi-like 2 *and − *3***	**Relative risk (RR)**	**95**% **CI**	**p-value**
n all low	22	**5.23**	1.67–16.32	**0.004**	15	**5.90**	1.33–26.23	**0.02**
n one gene elevated	24	**4.47**	1.47–13.61	**0.008**	26	2.84	0.77–10.41	0.12
n two genes elevated	22	reference			16	reference		

Significant p-values and relative risks are marked in bold face (p-values <0.05 were considered significant).

Finally, we analyzed the combined expression of the *Piwi-like 2–4* genes and their correlation with tumor-specific survival in a sex-specific manner and observed that different expression profiles were significantly correlated with survival in male and female patients. For male patients, decreased expression of *Piwi-like 2/Piwi-like 3* was correlated with a 5.9-fold increase in the risk of tumor-related death (95% CI: 1.33–26.23; p = 0.02). In female patients, decreased *Piwi-like 2/Piwi-like 4* expression was correlated with a 5.23-fold increase in the risk of tumor-related death (95% CI: 1.67–16.32; p = 0.004; Table 3 Figure 1). The results revealed that the combined expression of *Piwi-like* genes is correlated with tumor-specific survival in a sex-specific manner.

## Discussion

Previously we found that elevated levels of *Piwi-like 1* in STS and high as well as low levels of *Piwi-like 1* in pancreatic adenocarcinomas were significantly associated with worse patient’s survival. [18,19]. In our study, the levels of *Piwi-like 2–4* mRNA in STS were analyzed and correlated with each other, with clinical and histopathologic parameters and with tumor-specific survival. We found a significant correlation between tumor-specific survival and *Piwi-like 2 and Piwi-like 4* mRNA expression levels, but not with *Piwi-like 3* gene expression. There were correlations between the mRNA expressions of the *Piwi-like* genes and other stem cell-associated genes, such as *NanogOct3/4* and *survivin*. Surprisingly, a low expression of *Piwi-like 2* or *Piwi-like 3* was significantly correlated with a high expression level of *Nanog* and *Piwi-like-2* in addition with an increased transcript level of *Oct3/4*, but with a decreased level of the *survivin* 2B splice variant. Furthermore, low *Piwi-like 4* mRNA expression was correlated with a high level of the survivin Delta 3 splice variant. However, the survivin 2B splice variant is considered to be a pro-apoptotic protein, whereas the *survivin* Delta 3 splice variant has an anti-apoptotic function [22]. PIWIL2 protein may inhibit apoptosis via activation of Stat3/Bcl-X signaling [20].

A somewhat surprising result of our study was that a low mRNA expression level of all three *Piwi-like* genes was correlated with a poor prognosis, although higher protein levels have been reported to be associated with a poor survival [8-11]. There are several possible explanations for this phenomenon. Low mRNA transcription levels may characterize more aggressive tumors. For example, low mRNA levels for the oncogenes *mdm2* and *c-myc* have been correlated with a poor prognosis of patients with STS and squamous carcinomas of the tongue, respectively [23,24]. Another possibility is that there could be a negative autoregulatory feedback loop between protein and RNA levels in *Piwi-like* genes. Such a feedback loop has been described for other genes, for example for Snail1. Snail1 is a transcriptional repressor that binds to its own promoter and controls its expression [25]. Analogously, one may speculate that Piwi-like proteins, as the main effector component of the RNA-induced silencing complex (RISC), may regulate their own expression by post-transcriptional gene silencing via an unknown small RNA binding partner. Furthermore, different alternative transcripts leading to shortened protein products of PIWIL2 have been described recently [12]. The predominant form, PL2L60, is mainly expressed in tumor cells and promotes cell survival and proliferation of a human breast cancer cell line (MDA-MB-231), possibly by the upregulation of Bcl2 and Stat3. Because our primers targeted the 5′ end of the different mRNA transcripts for a better discrimination between the homologous genes, we were not able to measure the shortened transcript. Further studies are needed to determine if shortened alternative mRNA transcripts and protein isoforms for Piwi-like 3 and Piwi-like 4 also exist. Additionally, shorter transcripts and/or their protein products could decrease the levels of their full-length transcripts.

Piwi-like proteins bind exclusively to Piwi-interacting RNA (piRNA), a class of small RNAs that differ structurally from the classic siRNA and miRNA in several ways, including length (24–30 nt instead of 18–23 nt), the carriage of a 2′O-methyl modification at the 3′ end and their low conservation among even closely related species [26-28]. piRNAs are transcribed from a limited set of clusters located in pericentromeric or telomeric heterochromatic regions by an as yet unknown mechanism [29]. Their maturation is involved in a set of degradation steps involving transposon transcripts. Uncontrolled transposon activation in *Piwi* gene mutants has been observed in *Drosophila* and in mice [30,31], which points to a role of *Piwi-like* genes in transposon control during spermatogenesis. However, the mechanisms and consequences of *Piwi-like* gene re-initiation in tumors are not yet fully understood. Given our observation that a low mRNA expression of *Piwi-like* genes is correlated with increased tumor size and worse survival, one may speculate that a decrease in the mRNA of *Piwi-like* genes can derepress transposon activity and enhance tumor cell selection to a more proliferative, active and aggressive phenotype.

PIWI proteins appear to have sex-specific functions, especially in vertebrate male germ cell maturation [30-32]. We were able to show for the first time a sex-specific effect of *Piwi-like* gene expression on the survival of tumor patients. The presence of androgen and estrogen receptors has been reported in soft tissue sarcomas [33]. An in-silico search for binding sites for sex-steroid receptors in the putative promoter regions of the *Piwi-like* genes showed an androgen receptor binding site in the *Piwi-like 2* gene promoter (unpublished results). However, the transcriptional activation of the *Piwi-like* genes may also be initiated indirectly by transcription factors regulated by sex-steroid binding factors. This question should be addressed in further studies.

## Conclusions

In conclusion, a correlation of *Piwi-like 2–4* mRNA expression with tumor size and of *Piwi-like 2* and *Piwi-like 4* transcript levels with tumor-related death was found for STS patients. Additionally, there was a sex-specific association of the combination of low mRNA transcript levels for *Piwi-like 2/-3* and *Piwi-like 2/-4* with tumor-specific survival in male and in female STS patients, respectively. A low mRNA expression level for *Piwi-like 2–4* genes defines a significantly increased risk for tumor-related death and may have potential as a predictor of survival for both all STS patients and in a sex-specific manner.

## Competing interests

The authors report no potential conflicts of interest.

## Authors’ contributions

TG designed the study, analyzed the data and drafted the manuscript. FK performed experimental procedures, carried out molecular biological studies and analyzed the data, MKa and MBa aided in study design, analyzed the data and reviewed the manuscript. PW treated the patients, collected tumor/normal tissues and clinical data and participated in the study design, CL and SG performed and reviewed statistical analysis, HJH performed histo-pathological evaluation and reevaluation of sarcoma tissues, SW carried out molecular biological studies and analyzed the data, HT designed the study, analyzed the data and drafted the manuscript. All authors read and approved the final manuscript.

## Pre-publication history

The pre-publication history for this paper can be accessed here:

http://www.biomedcentral.com/1471-2407/12/272/prepub

## Supplementary Material

Additional file 1 **Figure S1** Binding sites of the commercial TaqMan primer in the different *Piwi-like* genes. Click here for file

Additional file 2 **Table S1** Spearman-Rho bivariate linear correlation analyses: Significant correlations of Piwi-like 2–4 mRNA expression with tumor size and gene expression levels of other stem cell-associated genes in our cohort of STS. Click here for file

Additional file 3 **Figure S2** Distribution of Piwi-like 2, -3, and −4 mRNA expression separated by gender. Click here for file
