# Supplementary material for: Expression of human Piwi-like genes is associated with prognosis for soft tissue sarcoma patients
Source: BMC Cancer. 2012 Jun 29;12:272. doi: 10.1186/1471-2407-12-272 (PMC3472179; doi:10.1186/1471-2407-12-272)
Supplement: Additional file 1 — Figure S1 Binding sites of the commercial TaqMan primer in the different Piwi-like genes. [file 1471-2407-12-272-S1.doc]

**Supplemental Figure 1: Binding sites of the commercial TaqMan primer in the different *Piwi-like* genes**

***Piwi-like 2***

5‘UTR

3‘UTR

Hs01032719_m1

5‘UTR

Hs00908837_m1

3‘UTR

13 14

4 5 6

***Piwi-like 3***

Hs00895218_m1

3‘UTR

1 2

***Piwi-like 4***
