# Supplementary material for: Expression of human Piwi-like genes is associated with prognosis for soft tissue sarcoma patients
Source: BMC Cancer. 2012 Jun 29;12:272. doi: 10.1186/1471-2407-12-272 (PMC3472179; doi:10.1186/1471-2407-12-272)
Supplement: Additional file 2 — Table S1 Spearman-Rho bivariate linear correlation analyses: Significant correlations of Piwi-like 2–4 mRNA expression with tumor size and gene expression levels of other stem cell-associated genes in our cohort of STS. [file 1471-2407-12-272-S2.doc]

**Supplemental Table 1: Spearman-Rho bivariate linear correlation analyses: Significant correlations of *Piwi-like 2-4* mRNA expression with tumor size and gene expression levels of other stem cell-associated genes in our cohort of STS**

| ***Piwi-like 2*** | **Parameter** | **Correlation** | **r_s_** | **n** |
| --- | --- | --- | --- | --- |
|  | Tumor size | 0.018 | 0.21 | 125 |
|  | *Survivin_2b* mRNA expression | 0.048 | 0.20 | 93 |
|  | *Oct4* mRNA expression | 0.002 | -0.31 | 94 |
|  | *Nanog* mRNA expression | 0.029 | -0.23 | 91 |
|  | *Piwi-like 4* mRNA expression | 0.0000297 | 0.36 | 125 |
|  |  |  |  |  |
| ***Piwi-like 3*** |  |  |  |  |
|  | Tumor size | 0.024 | 0.20 | 125 |
|  | *Nanog* mRNA expression | 0.02 | -0.24 | 91 |
|  |  |  |  |  |
| ***Piwi-like 4*** |  |  |  |  |
|  | Tumor size | 0.041 | 0.18 | 125 |
|  | *Nanog* mRNA expression | 0.02 | -0.24 | 91 |
|  | *Survivin D3* mRNA expression | 0.047 | -0.18 | 120 |
|  | *Piwi-like 2* mRNA expression | 0.0000297 | 0.36 | 125 |
|  |  |  |  |  |
