# Supplementary material for: Expression of human Piwi-like genes is associated with prognosis for soft tissue sarcoma patients
Source: BMC Cancer. 2012 Jun 29;12:272. doi: 10.1186/1471-2407-12-272 (PMC3472179; doi:10.1186/1471-2407-12-272)
Supplement: Additional file 3 — Figure S2 Distribution of Piwi-like 2, -3, and −4 mRNA expression separated by gender. [file 1471-2407-12-272-S3.doc]

**Supplemental Figure 2: Distribution of Piwi-like 2, -3, and -4 mRNA expression separated by gender**
